# Lance-Adams Syndrome Treated by Perampanel in the Acute Term

**DOI:** 10.7759/cureus.13761

**Published:** 2021-03-08

**Authors:** Masahito Katsuki, Norio Narita, Iori Yasuda, Teiji Tominaga

**Affiliations:** 1 Neurosurgery, Tohoku University, Sendai, JPN; 2 Neurosurgery, Kesennuma City Hospital, Kesennuma, JPN

**Keywords:** cardiopulmonary arrest, hypoxia, lance-adams syndrome, myoclonus, n-isopropyl-p-[123i]-iodoamphetamine single-photon emission computed tomography, perampanel, 3-dimensional stereotactic surface projections

## Abstract

Lance-Adams syndrome (LAS) is chronic post-hypoxic myoclonus after a hypoxic encephalopathy. Recently, the report on LAS in the chronic term treated by perampanel (PER) is increasing. However, PER’s efficacy in the “acute term” has not been reported. Here, we report an LAS patient who markedly improved when PER was added to his existing treatment regime in the acute term. The 65-year-old patient presented with a return of spontaneous circulation after cardiopulmonary arrest. He developed myoclonus on the admission day, and it led to tonic-clonic convulsion. We started levetiracetam 3000 mg/day, lacosamide 400 mg/day, general anesthesia using midazolam 180 mg/day, dexmedetomidine 1000 μg/day, and fentanyl 1.2 mg/day. We could stop the convulsions after 18 h from the onset. We tried to reduce sedatives, but his convulsion recurred. We added PER 2 mg/day for three days, PER 4 mg/day for next four days, then used PER 8 mg/day and we could gradually reduce the sedatives. Single-photon emission computed tomography on day 40 showed cerebral blood flow (CBF) increase at the bilateral anterior lobes of the cerebellum, medial temporal lobes, and supplementary motor and premotor areas, while CBF decrease at the brain surface of the frontal, parietal, and temporal lobes. The myoclonus disappeared since day 12, and he was transferred to another rehabilitation hospital on day 56. The optimal treatment strategy has not been established for LAS, but our case suggested that PER could be one of the choices to treat LAS in the acute term.

## Introduction

Lance-Adams syndrome (LAS) is chronic post-hypoxic myoclonus with onset days to weeks after a hypoxic encephalopathy [1,2]. LAS originated from a 1963 report in which four patients with acute hypoxic encephalopathy due to cardiac arrest after anesthesia or postoperative airway obstruction developed generalized myoclonus during recovery from coma [2]. The LAS’s myoclonus is variably associated with dysarthria, ataxia, seizures, or cognitive deficits [1]. Given LAS’s relative rarities, there are no controlled treatment studies of LAS. The majority of cases require polypharmacy management or general anesthesia therapy, with an incomplete response [1].

Recently, the report on LAS in the chronic term treated by perampanel (PER) is increasing [3-8]. However, PER’s efficacy in the acute term has not been reported. Here, we report an LAS patient who markedly improved when PER was added to his existing treatment regime in the acute term.

## Case presentation

A 65-year-old man, who did not have any past histories, suddenly fainted. He went into cardiopulmonary arrest (CPA) with bystanders, but the bystanders did not perform resuscitation. After 11 minutes, the ambulance and rescue team arrived and started resuscitation. After five cycles of chest compressions and rescue breaths at a ratio of 30:2, a return of spontaneous circulation was achieved, and the patient arrived at our hospital. His blood pressure was 158/108 mmHg, his heart rate 122 bpm, respiratory rate 15/min, blood temperature 36.8°C, and saturation of percutaneous oxygen 92% with 10 L/min oxygen. His consciousness was as Japan Coma Scale (JCS) 200 and Glasgow Coma Scale (GCS) 3 (E1VTM1). The pupil sizes were 5 mm equally in both eyes, and the eyes were roving. Laboratory tests reveal slight changes after resuscitation, but nothing related to sudden CPA. Echocardiography, electrocardiography, and coronary angiography did not suggest cardiologic diseases. Head computed tomography (CT) angiography and magnetic resonance imaging (MRI) also did not show any findings related to the CPA. The cause of CPA was unknown, and general care with ventilator and vasopressors was started.

**Figure 1:**
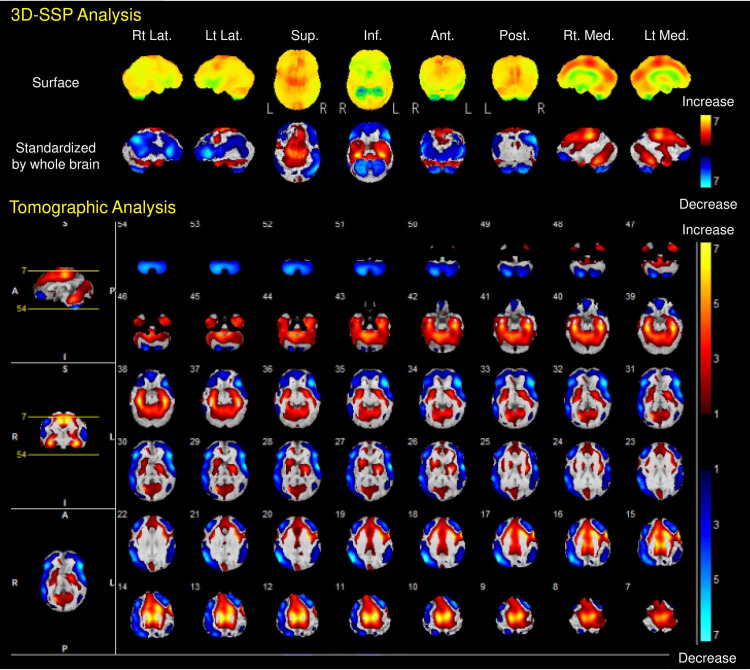
N-isopropyl-p-[123I]-iodoamphetamine single-photon emission computed tomography evaluated by 3-dimensional stereotactic surface projections (3D-SSP) standardized by the whole brain The cerebral blood flow (CBF) increased at the bilateral anterior lobes of the cerebellum, medial temporal lobes, and supplementary motor and premotor areas; while CBF decreased at the brain surface of the frontal, parietal, and temporal lobes. Ant: anterior; Inf: inferior; Lat: lateral; Med: medial; Post: posterior; Sup: superior.

After 4 hours from the onset, he presented 1 Hz left upper extremities’ myoclonus seizure intermittently lasting for around 3 minutes, and we started levetiracetam (LEV) 3000 mg/day, but the myoclonus could not be stopped. After 16 hours from the onset, we added lacosamide (LCM) 400 mg/day. However, his myoclonus continued. The next day, he developed tonic-clonic convulsions. We started general anesthesia with midazolam 180 mg/day, dexmedetomidine 1000 μg/day, and fentanyl 1.2 mg/day with adequate noradrenaline and dobutamine for about 48 h. Finally, we could stop the convulsions. We tried to reduce sedatives, but his convulsion or myoclonus recurred. Therefore, we added PER 2 mg/day on day 3. We performed a tracheostomy on day 5. On day 7, we prescribed PER 4 mg/day because we could not reduce the sedatives. We then used PER 8 mg/day from day 12, and we could gradually reduce the sedatives. Because of the circulatory failure and sepsis, we carefully and slowly reduced sedatives and vasopressors’ dosage. On day 38, we finally stopped sedatives and vasopressors. On day 39, we performed CT and MRI including diffusion tensor imaging, but they did not reveal any characteristic findings. On day 40, N-isopropyl-p-[123I]-iodoamphetamine single-photon emission computed tomography (123I-IMP-SPECT) was performed and evaluated by 3-dimensional stereotactic surface projections (3D-SSP) standardized by the whole brain [9]. The cerebral blood flow (CBF) increased at the bilateral anterior lobes of the cerebellum, medial temporal lobes, and supplementary motor and premotor areas; while CBF decreased at the brain surface of the frontal, parietal, and temporal lobes (Figure 1). We performed electroencephalography (EEG), but only noise due to alternating current was detected despite using an electromagnetic noise prevention sheet and thiopental sedation. His consciousness improved as JCS 3 and GCS 10 (E4VTM6), and rehabilitation was continued using a wheelchair. The myoclonus has disappeared since day 12, and he was transferred to another rehabilitation hospital on day 56 because he is from another prefecture.

## Discussion

There are no well-defined guidelines for treatment in LAS. Our case presented status epilepticus started from myoclonic seizures on the admission day. We used multiple antiepileptic drugs and performed general anesthesia therapy according to the Japanese guideline [10]. We could not reduce sedative drugs without PER, but after adding PER, the patient was extubated and started rehabilitation. Although the general anesthesia therapy’s effect should be considered, our case suggested the efficacy of PER for LAS’s myoclonic seizures and subsequent status epilepticus as the acute treatment choice.

Lance-Adams syndrome

The LAS’s myoclonic seizure mechanism is unclear but hypothesized to be originated from the cortex, and the myoclonus may relate to abnormal gamma-aminobutyric acid (GABA) and serotonin neurotransmission in the brain [1,4,8]. We did not evaluate GABA or serotonin levels in serum and cerebrospinal fluid, because we could not order these tests in our hospital. However, we speculate that the patient’s GABA and serotonin neurotransmission system is dysregulated as previously reported hypothesis.

Previous reports describe that SPECT of LAS patients showed decreased blood flow in the right basal ganglia and left temporal lobe [11,12]. Ours showed CBF increase at the bilateral supplementary motor and premotor areas as well as CBF decrease at the brain surface of the frontal, parietal, and temporal lobes (Figure 1). This is different findings compared to the previous reports because the region where the CBF was elevated was different from the previous reports [11,12]. Previous functional imaging study demonstrated increased glucose metabolism in the ventrolateral thalamus, pontine tegmentum, and medial temporal lobes, suggesting an involvement of the basal ganglia-thalamo-cortical network [13,14]. The ventrolateral thalamus connects to the motor cortex, premotor cortex, and supplementary motor cortex [15]. These cortexes play important roles in initiating motor function. Our SPECT results suggested that CBF might increase at the medial frontal lobes due to increased glucose metabolism in the ventrolateral thalamus, causing myoclonus. CBF decrease at the whole brain surface may be due to results of hypoxia. Further CBF and functional studies are needed.

There are no typical findings of LAS’s EEG [1,11]. In the literature review, burst suppression was the most common pattern, while alpha coma was the least common pattern [1]. We performed EEG, but only noise due to alternating current was detected despite using an electromagnetic noise prevention sheet and thiopental sedation. This might be a new characteristic EEG finding of LAS, but further studies are needed.

LAS’s somatosensory evoked potentials (SEP) sometimes show characteristic findings. The early SEP component is high amplitude so-called a giant SEP. Then, C-reflex, which records muscle activity at long latency after electrical stimulation, can be detected [4,8]. Oi et al. showed a positive correlation between PER blood concentration and prolongation of latency, such as P25 in giants SEP, which can be used as a potential biomarker for assessing the objective effects of PER on intractable cortical myoclonus [8]. We did not perform SEP evaluation because we do not have these instruments nor neurologist who could evaluate the results, and we should have performed it before and after PER prescription to evaluate PER’s efficacy.

Perampanel

Perampanel (PER) is a selective noncompetitive α-amino-3-hydroxy-5-methyl-4-isoxazole propionic acid (AMPA) receptor antagonist and inhibits excessive excitation of nerve cells by reducing Ca2+ inflow. PER was recently introduced as adjunctive therapy for patients with epilepsy [4,8,16]. The pharmacological characteristics of PER - (1) dose-dependent reduction of seizure and myoclonus [17]; (2) possible threshold dosage, e.g., 6 mg/day (maximum dose of PER is generally 12 mg/day) for severe side effects, such as irritability, anxiety, violence, hallucination, weight gain [18]; (3) very slow titration was recommended to avoid side effects [16] - are reported. While PER shows promise as an antiepileptic drug with a new mechanism, it must be used cautiously due to side effects. In our case, the myoclonus and the subsequent status epilepticus could be stopped by using 8 mg/day PER with a gradual increase in the short term, but we should pay attention to side effects. We will reduce PER to around 2-6 mg/day, according to the previous report [8,18].

Table 1 shows the previously reported LAS treated by PER. In all previous reports, PER is prescribed for chronic LAS, and their myoclonus or activities in daily livings were improved [3-8]. We prescribed PER for LAS in the acute phase, and we could stop sedation and reach extubation. It is unknown whether the status epilepticus resolved by prolonged sedation or PER, but our case suggested that PER could be one of acute treatments for LAS. Further studies are desired on LAS and PER.

**Table 1:** Previous reports of Lance-Adams syndrome patients treated with perampanel ACET: Acetazolamide; CBZ: Carbamazepine; CLON: Clonazepam; LAC: Lacosamide; LEV: Levetiracetam; NA: Not Available; PER: Perampanel; PIR: Piracetam; PRIM: Primidone; VAL: Sodium valproate; ZNS: Zonisamide; 5-HT: 5-hydroxytryptophan.

Case No.	Author	Year	Age	Sex	Antecedent event	Duration of LAS prior to PER (years)	Medication treatment prior to PER (mg/d)	Perampanel dose (mg/d)	Clinical response	Drugs after adding PER	Follow-up period	Adverse effects
1	Steinhoff et al. [5]	2016	36	M	Cardiac arrest due to Brugada syndrome	1	LEV (2000); VAL (1500); CLON (2); PIR (7600); LAC (100)	2 for 3 days, then 4	Almost complete cessation of myoclonus at 4 mg/d	PIR and LAC stopped	>4 weeks	Somnolence
2	Lazaro et al. [6]	2017	35	M	Cardiac arrests	NA	LEV; VAL; Propofol; Thiopental; PIR; ZNS; Clonidine; Sodium oxybate; 5-HT; Gabapentin	24	Controlled the myoclonus	Only LEV, gabapentin, PER, and risperidone were continued	NA	Behavioral disorders
3	Yelden et al. [7]	2019	69	M	Severe pneumonia	NA	LEV; VAL; CLON	NA	Myoclonus improved with improved function	CLON reduced	NA	NA
4	Yelden et al. [7]	2019	37	F	Decannulation of the tracheostomy tube	NA	LEV; VAL; CLON	NA	Improvement of ambulation and speech	CLON and VAL stopped	NA	NA
5	Oi et al. [8]	2019	47	M	NA	NA	LEV; CLON; PRIM; CBZ; PIR	10	Myoclonus improved from marked to severe; ADL improved	NA	NA	None
6	Oi et al. [8]	2019	31	M	NA	NA	CLON; PIR	4	No improvement in myoclonus; ADL improved	NA	NA	Dizziness and palpitation
7	Lim et al. [3]	2020	63	M	Cardiac arrest in the postoperative period	6	LEV (1000); VAL (400); CLON (1); ACET (250)	2 for a week, then 4	Bouncy gait improved	Not reduced	6 weeks	No
8	Saito et al. [4]	2020	49	M	Severe bronchial asthma attack	11	PIR (15000); CLON (3.75); CBZ (600); baclofen (20); etizolam (0.5); alprazolam (0.8); PRIM (500); diazepam (6); Tokishakuyakusan (7500); LEV (2500)	2 to 10	Myoclonus improved with improved function	PIR (20); CLON (1.5); LEV (2000)	4 years	No
9	Our case	2021	65	M	Seizure followed by choking	Acute term	LEV (3000); LCM (400); midazolam (180); dexmedetomidine (1); fentanyl (1.2), with adequate noradrenaline and dobutamine.	2 for 3 days, 4 for 4 days, then 8	Improvement of myoclonus and consciousness	Withdrawal of general anesthesia, consciousness improved	56 days	No

## Conclusions

We herein reported an LAS patient who improved his myoclonus and subsequent status epilepticus markedly when PER was added to his existing treatment regime in the acute term. The optimal treatment strategy has not been established, but our case suggested that PER could be one of the choices to treat LAS in the acute term.
